# Lurasidone, olanzapine, and quetiapine extended‐release for bipolar depression: A systematic review and network meta‐analysis of phase 3 trials in Japan

**DOI:** 10.1002/npr2.12137

**Published:** 2020-09-09

**Authors:** Taro Kishi, Reiji Yoshimura, Kenji Sakuma, Makoto Okuya, Nakao Iwata

**Affiliations:** ^1^ Department of Psychiatry Fujita Health University School of Medicine Toyoake Japan; ^2^ Department of Psychiatry University of Occupational and Environmental Health Kitakyushu Japan

**Keywords:** bipolar depression, efficacy/safety/tolerability, lurasidone, olanzapine, quetiapine extended‐release, systematic review and network meta‐analysis

## Abstract

**Aim:**

This systematic review and random‐effect model, network meta‐analysis of the phase 3 trials in Japan assessed the efficacy and safety profile of lurasidone compared with olanzapine and quetiapine extended‐release (QUE‐XR) for the treatment of bipolar depression.

**Methods:**

The study included double‐blind, randomized, placebo‐controlled, phase 3 trials in Japan that included patients with bipolar depression. Outcomes included response rate (primary), remission rate (secondary), improvement of Montgomery‐Åsberg Depression Rating Scale (MADRS) total score, discontinuation rates, and incidence of individual adverse events.

**Results:**

Three studies were included (n = 1223). Lurasidone and olanzapine but not QUE‐XR were superior to placebo in response rate [risk ratio (95% credible interval): lurasidone = 0.78 (0.66, 0.92); olanzapine = 0.84 (0.71, 0.99); QUE‐XR = 0.87 (0.73, 1.03)]. Lurasidone, olanzapine and QUE‐XR were superior to placebo in remission rate [lurasidone = 0.90 (0.83, 0.98); olanzapine = 0.87 (0.77, 0.99); QUE‐XR = 0.84 (0.73, 0.98)] and the improvement of MADRS total score. There were not differences in discontinuation rates between each antipsychotic and placebo. Compared with placebo, lurasidone was higher incidence of akathisia, and increased body weight and blood prolactin level; olanzapine was higher incidence of somnolence and ≥7% weight gain, and increased body weight, blood total cholesterol level, blood LDL cholesterol level, and blood triglyceride levels; QUE‐XR was higher incidence of extrapyramidal symptoms, akathisia, somnolence, dry mouth, constipation and ≥7% weight gain, and increased body weight, blood total cholesterol level, blood LDL cholesterol level, and blood triglyceride levels.

**Conclusions:**

Our results suggested although the efficacy of three SGAs was similar, there were the differences in the safety profile among the SGAs.

## Abstract 2

The efficacy of three SGAs was similar. The safety profile was different in three SGAs.

## Graphical abstract

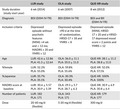

## 1 INTRODUCTION

Bipolar disorder (BD) is a common chronic psychiatric disorder, with a worldwide prevalence of approximately 1%.^1^ According to the Diagnostic and Statistical Manual of Mental Disorders, Fifth Edition, the diagnosis of bipolar I disorder (BDI) requires only the occurrence of a manic episode; however, the diagnosis of bipolar II disorder (BDII) requires at least one distinct episode of hypomania and one distinct episode of major depression during a patient's lifetime. More than 70% of suicide deaths and suicide attempts in patients with BD occur during depressive phase.^2^

In Japan, olanzapine^3^ and quetiapine extended‐release (QUE‐XR),^4^ followed by lurasidone^5^ in 2020 for use in the treatment of bipolar depression (BDep), were approved in Japan. Which second generation antipsychotics (SGAs) is the best drug for the treatment of Japanese patients with BDep? The most recent guideline recommends quetiapine, lithium, lamotrigine, and lurasidone are all recommended as first‐line treatment options with evidence for efficacy as monotherapy for the treatment of BDep.^2^ However, our question remained unanswered because there is difference in the approval dose of lurasidone for BDep between Japan (20‐60 mg/d) and other countries (20‐120 mg/d). Therefore, we conducted a systematic review and network meta‐analysis of the Japan phase 3 studies of these SGAs for the patients with BDep to investigate whether there were differences in efficacy, safety, and tolerability for the treatment of those patients among the SGAs.

## 2 METHODS

This study was performed according to the Preferred Reporting Items for Systematic Reviews and Meta‐Analyses guidelines (Table S1).^6^ The literature search, data extraction, and data input into spreadsheets for analysis were done simultaneously and independently by at least two authors (TK, KS and MO). The authors double‐checked the accuracy of data transfer and calculations in the study.

### 2.1 PICO search

Patients with BDep who were not being treated with any mood stabilizers or antipsychotics at the baseline were eligible. The intervention groups were administered lurasidone 20‐60 mg/d or olanzapine 5‐20 mg/d or QUE‐XR 300 mg/d (we included drugs with approved drugs and approved doses for the treatment of BDep in Japan), and the control group was administered placebo. The outcomes were efficacy and safety/tolerability (detailed information in the following section).

### 2.2 Literature search

We included only double‐blind, randomized, placebo‐controlled trials (DBRPCTs). Relevant studies were independently identified by the authors through searches of Embase, PubMed, and Cochrane Library, without language restrictions, from the date of inception of these databases to May 22, 2020. The following search strategy keywords were used: (random*) AND (Japan*) AND (bipolar OR bipolar depression) AND (placebo). Additional searches were conducted of ClinicalTrials.gov (http://clinicaltrials.gov/), the ISRCTN registry (https://www.isrctn.com/), UMIN Clinical Trials Registry (https://www.umin.ac.jp/ctr/index.htm), and the International Clinical Trials Registry Platform (http://www.who.int/ictrp/en/). We also performed a hand search to identify any other articles. Ultimately, three DBRPCTs met the criteria and were included in the present systematic review.

### 2.3 Data extraction and data synthesis

Intention‐to‐treat or modified intention‐to‐treat data were used in the analysis. We included outcomes that reported data from all three selected DBRPCTs. Outcomes were response rate (primary outcome, all studies defined response as ≥50% reduction in Montgomery‐Åsberg Depression Rating Scale (MADRS)^7^ at endpoint), remission rate (secondary outcome, two studies^3^, ^5^ defined remission as MADRS total score ≤ 8 and one study^4^ defined remission as a MADRS total score ≤ 12), improvement of MADRS total score, discontinuation rates, and incidence of individual adverse events. The methodological qualities of the included articles were assessed according to the Cochrane risk‐of‐bias tool.^8^

### 2.4 Meta‐analysis methods

A Bayesian network meta‐analysis based on random‐effects models^9^ was conducted using the netmeta package.^10^ Risk ratios (RRs) and standardized mean differences (SMDs) and their 95% credible intervals (95% Crls) were calculated for dichotomous data and continuous data, respectively. For cases where the RRs showed statistically significant between‐group differences with respect to treatment efficacy, discontinuation rates, or the incidence of individual adverse events based on RRs were significant, either the number needed to treat to benefit (NNTB) or the number needed to treat to harm (NNTH) was calculated from the risk difference (RD), using the formula NNTB or NNTH = 1/RD. We did not explore the heterogeneity, the consistency, and publication bias because only one study was included in each treatment group. Therefore, although we incorporated results into the Confidence in Network Meta‐Analysis application to assess the credibility of findings from network meta‐analysis,^11^ the confidence in the evidence for all outcomes was very low.

## 3 RESULTS

### 3.1 Study characteristics

The result of literature search was shown Figure S1. The search identified three DBRPCTs.^3^, ^4^, ^5^ Three studies were sponsored by pharmaceutical companies. Lurasidone study was unpublished. The data of lurasidone study are published at ClinicalTrials.gov (NCT01986101).^5^ The methodological quality of two studies was high as assessed with the Cochrane risk‐of‐bias tool (Figure S2). The study and patient characteristics are presented in Table 1. Although QUE‐XR study included BDI and BII patients, other two studies included only BDI patients. Although study duration of QUE‐XR study was 8 weeks, that of other two studies were 6 weeks. The results of meta‐analysis were shown Tables 2 and 3.

**Table 1:** Double‐blind, randomized, placebo‐controlled, phase 3 trials for bipolar depression included in the analysis

	LUR study	OLA study	QUE‐XR study
Study duration (study start year)	6 wk (2014)	6 wk (2007)	8 wk (2012)
Diagnosis	BDI (DSM‐IV‐TR)	BDI (DSM‐IV‐TR)	BDI and BII (DSM‐IV‐TR)
Inclusion criteria	Depressed episode without psychotic features (MINI): ≥4 wk and < 12 mo, MADRS ≥ 20 and YMRS ≤ 12	Depressed episode: ≤90 d at the time of randomization, HRSD‐17 ≥ 18 and YMRS < 8	Depressed episode (MINI), HRSD‐17 ≥ 20 and a HRSD‐17 depressed mood score ≥ 2 points and YMRS ≤ 12
Age	LUR: 42.6 ± 12.9 PLA: 41.3 ± 12.6	OLA: 36.0 ± 11.1 PLA: 35.0 ± 11.0	QUE‐XR: 38.1 ± 11.2 PLA: 38.8 ± 11.0
%female	LUR: 52.2% PLA: 55.0%	OLA: 59.8% PLA: 55.6%	QUE‐XR: 52.0% PLA: 57.1%
%Japanese	LUR: 35.7% PLA: 35.1%	OLA: 30.3% PLA: 30.4%	QUE‐XR: 100% PLA: 100%
MADRS score at baseline	LUR: 30.6 ± 5.6 PLA: 30.9 ± 5.4	OLA: 29.3 ± 5.7 PLA: 28.7 ± 6.3	QUE‐XR: 30.9 ± 6.9 PLA: 30.8 ± 6.4
Number of patients	LUR: 182 PLA: 171	OLA: 343 PLA: 171	QUE‐XR: 179 PLA: 177
Dose	20‐60 mg/d (flexible)	5‐20 mg/d (flexible)	300 mg/d

Abbreviations: BD, bipolar disorder; DSM‐IV‐TR, Diagnostic and Statistical Manual of Mental Disorders, Fourth Edition, Text Revision; HRSD‐17, 17‐item Hamilton Rating Scale for Depression total score; LUR, lurasidone; MADRS, Montgomery‐Åsberg Depression Rating Scale total score; MINI, Mini‐International Neuropsychiatric Interview; OLA, olanzapine; PLA, placebo; QUE‐XR, quetiapine extended‐release; YMRS, Young Mania Rating Scale total score.

**Table 2:** Results of network meta‐analysis for dichotomous outcomes

		RR (95% CI)*	NNTB or NNTH (95% CI)*
Response rate	LUR vs PLA	**0.78 (0.66, 0.92)**	**6.6 (4.0, 19.6)**
	OLA vs PLA	**0.84 (0.71, 0.99)**	**10.9 (5.5, 1000.0)**
	QUE‐XR vs PLA	0.87 (0.73, 1.03)	
Remission rate	LUR vs PLA	**0.90 (0.83, 0.98)**	**11.4 (6.3, 55.6)**
	OLA vs PLA	**0.87 (0.77, 0.99)**	**10.9 (5.6, 142.9)**
	QUE‐XR vs PLA	**0.84 (0.73, 0.98)**	**8.8 (4.7, 55.6)**
All‐cause discontinuation	LUR vs PLA	0.77 (0.48, 1.22)	
	OLA vs PLA	0.77 (0.57, 1.05)	
	QUE‐XR vs PLA	0.94 (0.65, 1.37)	
Discontinuation due to adverse events	LUR vs PLA	0.80 (0.28, 2.34)	
	OLA vs PLA	1.15 (0.62, 2.15)	
	QUE‐XR vs PLA	1.67 (0.93, 2.99)	
Extrapyramidal symptoms	LUR vs PLA	0.94 (0.24, 3.68)	
	OLA vs PLA	1.58 (0.85, 2.94)	
	QUE‐XR vs PLA	**3.11 (1.36, 7.09)**	**12.0 (7.2, 37.0)**
Akathisia	LUR vs PLA	**2.04 (1.03, 4.04)**	**15.2 (7.9, 166.7)**
	OLA vs PLA	3.49 (0.80, 15.18)	
	QUE‐XR vs PLA	**3.71 (1.26, 10.95)**	**16.4 (9.3, 66.7)**
Dizziness	LUR vs PLA	0.53 (0.16, 1.79)	
	OLA vs PLA	1.08 (0.42, 2.79)	
	QUE‐XR vs PLA	4.94 (0.58, 41.)	
Somnolence	LUR vs PLA	0.94 (0.34, 2.61)	
	OLA vs PLA	**2.67 (1.44, 4.96)**	**9.3 (6.2, 18.9)**
	QUE‐XR vs PLA	**19.78 (7.40, 52.83)**	**2.4 (2.0, 2.9)**
Insomnia	LUR vs PLA	0.75 (0.20, 2.74)	
	OLA vs PLA	0.61 (0.26, 1.44)	
	QUE‐XR vs PLA	0.99 (0.02, 49.56)	
Dry mouth**	LUR vs PLA	1.87 (0.17, 20.43)	
	OLA vs PLA	1.50 (0.61, 3.70)	
	QUE‐XR vs PLA	**9.89 (4.04, 24.21)**	**4.0 (3.1, 5.5)**
Constipation	LUR vs PLA	0.94 (0.19, 4.57)	
	OLA vs PLA	1.89 (0.72, 4.99)	
	QUE‐XR vs PLA	**4.61 (1.35, 15.78)**	**16.4 (9.5, 55.6)**
Nasopharyngitis	LUR vs PLA	1.17 (0.47, 2.89)	
	OLA vs PLA	1.99 (0.83, 4.79)	
	QUE‐XR vs PLA	1.35 (0.78, 2.35)	
At least 7% weight gain	LUR vs PLA	4.64 (0.22, 95.90)	
	OLA vs PLA	**19.58 (4.87, 78.69)**	**4.5 (3.7, 5.8)**
	QUE‐XR vs PLA	**5.41 (1.22, 24.04)**	**19.6 (11.1, 83.3)**

Abbreviations: 95% CI, 95% credible interval; LUR, lurasidone; MADRS, Montgomery‐Åsberg Depression Rating Scale total score; NNTB or NNTH, number needed to treat to benefit or harm; ns, not significant; OLA, olanzapine; PLA, placebo; QUE‐XR, quetiapine extended‐release; RR, risk ratio.

* Boldface indicates statistical significance.

** RR (95% CI): LUR vs QUE‐XR = 0.19 (0.02, 2.43), NNTH (95% CI) = 4.1 (3.1, 5.8); OLA vs QUE‐XR = 0.15 (0.04, 0.54), NNTH (95% CI) = 4.3 (3.2, 6.5); LUR vs OLA = ns.

**Table 3:** Results of network meta‐analysis for continuous outcomes

		SMD (95% CI)*
MADRS score	LUR vs PLA	−**0.32 (**−**0.53,** −**0.11)**
	OLA vs PLA	−**0.25 (**−**0.44,** −**0.06)**
	QUE‐XR vs PLA	−**0.22 (**−**0.43,** −**0.02)**
Body weight**	LUR vs PLA	**0.27 (0.06, 0.48)**
	OLA vs PLA	**0.81 (0.62, 1.00)**
	QUE‐XR vs PLA	**0.67 (0.46, 0.89)**
Blood glucose level	LUR vs PLA	0.01 (−0.20, 0.23)
	OLA vs PLA	0.12 (−0.08, 0.31)
	QUE‐XR vs PLA	0.14 (−0.07, 0.35)
Blood HbA1c level	LUR vs PLA	0.14 (−0.07, 0.35)
	OLA vs PLA	0.05 (−0.15, 0.24)
	QUE‐XR vs PLA	0.00 (−0.21, 0.21)
Blood total cholesterol level***	LUR vs PLA	0.06 (−0.16, 0.27)
	OLA vs PLA	**0.36 (0.17, 0.55)**
	QUE‐XR vs PLA	**0.36 (0.15, 0.57)**
Blood LDL cholesterol level****	LUR vs PLA	0.04 (−0.17, 0.26)
	OLA vs PLA	**0.34 (0.15, 0.54)**
	QUE‐XR vs PLA	**0.27 (0.06, 0.48)**
Blood HDL cholesterol level	LUR vs PLA	−0.16 (−0.37, 0.06)
	OLA vs PLA	0.12 (−0.07, 0.31)
	QUE‐XR vs PLA	0.06 (−0.15, 0.27)
Blood triglyceride levels*****	LUR vs PLA	−0.11 (−0.32, 0.10)
	OLA vs PLA	**0.23 (0.05, 0.42)**
	QUE‐XR vs PLA	**0.38 (0.17, 0.59)**
Blood prolactin level	LUR vs PLA	**0.27 (0.05, 0.48)**
	OLA vs PLA	0.12 (−0.08, 0.31)
	QUE‐XR vs PLA	0.07 (−0.14, 0.28)

Abbreviations: 95% CI, 95% credible interval; LUR, lurasidone; MADRS, Montgomery‐Åsberg Depression Rating Scale total score; ns, not significant; OLA, olanzapine; PLA, placebo; QUE‐XR, quetiapine extended‐release; SMD, standardized mean difference.

* Boldface indicates statistical significance.

** SMD (95% CI): LUR vs OLA = −0.54 (−0.83, −0.26); LUR vs QUE‐XR = −0.41 (−0.71, −0.11); OLA vs QUE‐XR = ns.

*** SMD (95% CI): LUR vs OLA = −0.31 (−0.59, −0.02); LUR vs QUE‐XR = −0.31 (−0.61, −0.01); OLA vs QUE‐XR = ns.

**** SMD (95% CI): LUR vs OLA = −0.30 (−0.59, −0.01); LUR vs QUE‐XR: ns; OLA vs QUE‐XR = ns.

***** SMD (95% CI): LUR vs OLA = −0.35 (−0.63, −0.06); LUR vs QUE‐XR = −0.49 (−0.79, −0.19); OLA vs QUE‐XR = ns.

### 3.2 Efficacy

Lurasidone and olanzapine but not QUE‐XR outperformed placebo regarding response rate. The RR (95% Crl) and NNTB (95% Crl) of each SGA were as follows: lurasidone = 0.78 (0.66, 0.92) and 6.6 (4.0, 19.6); olanzapine = 0.84 (0.71, 0.99) and 10.9 (5.5, 1000.0); QUE‐XR = not significant. Lurasidone, olanzapine, and QUE‐XR outperformed placebo regarding remission rate. The RR (95% Crl) and NNTB (95% Crl) of each SGA were as follows: lurasidone = 0.90 (0.83, 0.98) and 11.4 (6.3, 55.6); olanzapine = 0.87 (0.77, 0.99) and 10.9 (5.6, 142.9); QUE‐XR = 0.84 (0.73, 0.98) and 8.8 (4.7, 55.6). Three SGAs also outperformed placebo regarding the improvement of MADRS total score. We did not find any differences in all efficacy outcomes among the SGAs.

### 3.3 Safety, tolerability, and adverse effects

There were not significant differences in discontinuation rates among all treatments. However, lurasidone was higher incidence of akathisia, and increased body weight and blood prolactin level compared with placebo. Olanzapine was higher incidence of somnolence and ≥7% weight gain, and increased body weight, blood total cholesterol, LDL cholesterol, and triglyceride levels compared with placebo. Quetiapine extended‐release was higher incidence of extrapyramidal symptoms, akathisia, somnolence, dry mouth, constipation and ≥7% weight gain, and increased body weight, blood total cholesterol, LDL cholesterol, and triglyceride levels compared with placebo. quetiapine extended‐release was higher incidence of dry mouth compared with lurasidone and olanzapine. Olanzapine and QUE‐XR increased body weight, blood total cholesterol, and triglyceride levels compared with lurasidone. Olanzapine increased blood LDL cholesterol level compared with lurasidone.

## 4 DISCUSSION

To the best of our knowledge, this is the first systematic review and network meta‐analysis of DBRPCTs of lurasidone, olanzapine, and QUE‐XR for the treatment of adult patients with BDep in Japan. The QUE‐XR trials included only patients from Japan, whereas the lurasidone and olanzapine trials were international studies. Although QUE‐XR did not outperform placebo regarding response rate, the effect size of three SGAs on efficacy outcomes when compared with placebo seemed to be similar. However, olanzapine and QUE‐XR had the risk of somnolence and metabolic syndrome. In addition, QUE‐XR had the risk of anticholinergic adverse events and extrapyramidal symptoms. On the other hand, although lurasidone had the risk of akathisia and increase body weight and blood PRL level, the effect size for lurasidone on these outcomes when compared with placebo was not large. Thus, the differences in the safety profile among three SGAs were observed.

There were several limitations to this study. First, this study included only three DBRPCTs. The confidence in the evidence for all outcomes was very low, and the external validity of analysis might be limited. Second, because the lurasidone and olanzapine trials included Japanese and non‐Japanese patients, the results may not directly reflect clinical practice in Japan. However, the number of Japanese patients in these trials was small. Third, we identified significant differences in some blood examination outcomes between the SGA and placebo groups. Because not all the trials had inclusion and exclusion criteria with respect to these outcomes, the baseline values might influence the results. Fourth, although concomitant medication might influence the results in each trial, because only lurasidone trial reported the incidence of concomitant drug use, we did not evaluate an association between this potential confounding factor and the results of our study.

Our results suggested although the efficacy of three SGAs was similar, there were the differences in the safety profile among the SGAs. It notes that the results of this study should be interpreted with consideration of the different study design characteristics of the trials.

## CONFLICT OF INTEREST

The present study was supported by the Grant‐in‐Aid for Scientific Research (C) (19K08082). The authors have declared that there are no conflicts of interest relating to the subject of this study. Interests from the past 3 years are as follows. Dr Kishi received speaker's honoraria from Daiichi Sankyo, Dainippon Sumitomo, Eisai, Janssen, Otsuka, Meiji, Mochida, MSD, and Tanabe‐Mitsubishi (Yoshitomi); as well as research grants from the Japanese Ministry of Health, Labour and Welfare, Grant‐in‐Aid for Scientific Research (C), and Fujita Health University School of Medicine. Prof. Yoshimura has received speaker's honoraria from Dainippon Sumitomo, Eli Lilly, Otsuka, and Meiji. Dr Sakuma has received speaker's honoraria from Eisai, Kissei, Meiji, Otsuka, and Torii; and has received a Fujita Health University School of Medicine research grant, as well as a Grant‐in‐Aid for Young Scientists (B). Dr Okuya has received speaker's honoraria from Meiji. Dr Iwata received speaker's honoraria from Astellas, Dainippon Sumitomo, Eli Lilly, GlaxoSmithKline, Janssen, Yoshitomi, Otsuka, Meiji, Shionogi, Novartis, and Pfizer; along with research grants from GlaxoSmithKline, Meiji, and Otsuka.

## AUTHOR CONTRIBUTIONS

TK was involved in the study concept and design and performed the statistical analysis. TK, KS, and MO performed acquisition and interpretation of the data. All the authors wrote the manuscript. NI and RY supervised the review.

## APPROVAL OF THE RESEARCH PROTOCOL BY AN INSTITUTIONAL REVIEWER BOARD

Not applicable.

## INFORMED CONSENT

Not applicable.

## REGISTRY AND THE REGISTRATION NO. OF THE STUDY/TRIAL

Not applicable.

## ANIMAL STUDIES

Not applicable.

## Supporting information

Fig S1‐S3

Table S1

## Data Availability

The data that support the findings of this study are openly available in the articles of three studies^3^, ^4^, ^5^ that cited in this paper.
