# Supplementary material for: Lurasidone, olanzapine, and quetiapine extended‐release for bipolar depression: A systematic review and network meta‐analysis of phase 3 trials in Japan
Source: Neuropsychopharmacol Rep. 2020 Sep 9;40(4):417–22. doi: 10.1002/npr2.12137 (PMC7722645; doi:10.1002/npr2.12137)

Supplementary Figure 1. Flow of the literature search


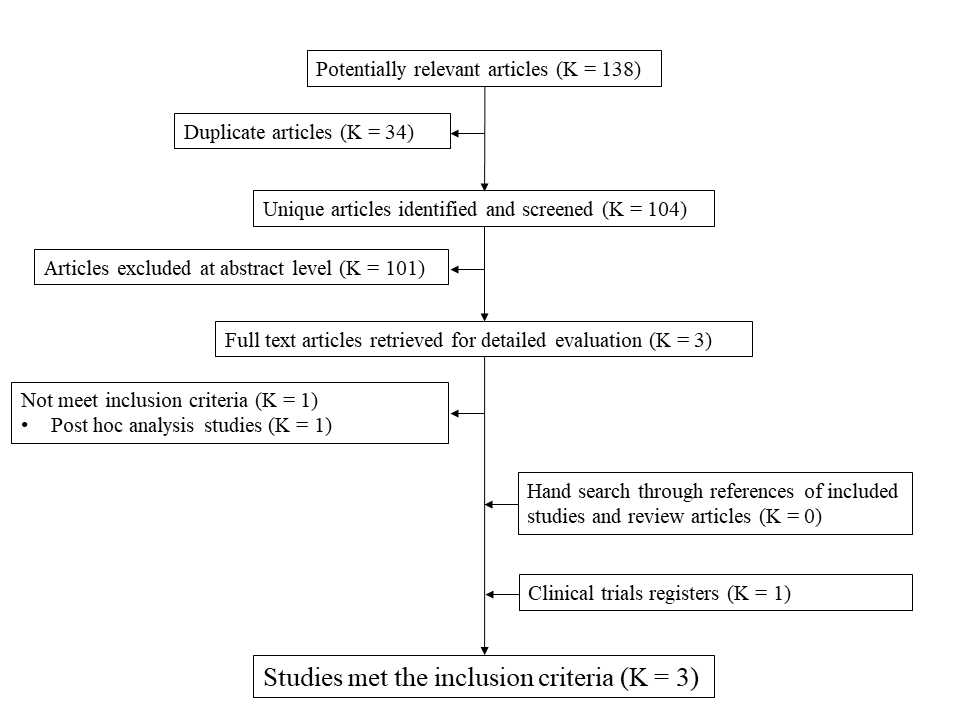


Supplementary Figure 2. Risk of bias summary


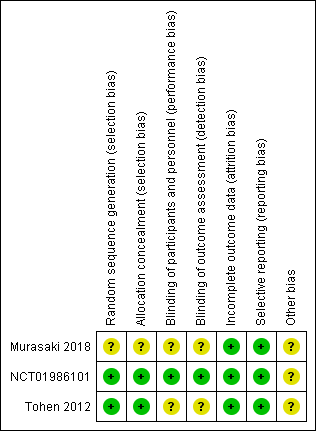

Supplement: Supplementary file 1 — Fig S1‐S3 [file NPR2-40-417-s001.docx]
